# Silodosin 8 mg improves benign prostatic obstruction in Caucasian patients with lower urinary tract symptoms suggestive of benign prostatic enlargement: results from an explorative clinical study

**DOI:** 10.1186/s12894-018-0326-7

**Published:** 2018-03-05

**Authors:** Ferdinando Fusco, Massimiliano Creta, Nicola Longo, Francesco Persico, Marco Franco, Vincenzo Mirone

**Affiliations:** Department of Neurosciences, Human Reproduction and Odontostomatology, University of Naples, Federico II - Via Pansini 5, 80131 Naples, Italy

**Keywords:** Benign prostatic enlargement, Benign prostatic obstruction, Lower urinary tract symptoms, Silodosin, Urodynamic

## Abstract

**Background:**

To preliminary investigate the effects of silodosin 8 mg once daily on obstruction urodynamic parameters and subjective symptoms in Caucasian patients with lower urinary tract symptoms suggestive of benign prostatic enlargement.

**Methods:**

We performed a single-center, open-label, single-arm, post-marketing interventional clinical trial. Inclusion criteria were: Caucasian subjects aged ≥50 years waiting to undergo surgery for lower urinary tract symptoms suggestive of benign prostatic enlargement, international prostate symptom total score ≥ 13, international prostate symptom-quality of life score ≥ 3, prostate volume ≥ 30 ml, maximum urine flow rate ≤ 15 mL/s, bladder outlet obstruction index > 40. Eligible subjects received one capsule of silodosin 8 mg once daily for 8 weeks. Invasive urodynamic evaluations were performed at baseline and at 8-weeks follow-up. International prostate symptom questionnaire was administered at baseline, after 4-weeks and 8-weeks of treatment.

**Results:**

Overall, 34 subjects were included. Mean bladder outlet obstruction index significantly decreased from 70.6 to 39.2 and bladder outlet obstruction index class improved in 16 patients (53.3%). Statistically significant improvements of mean total international prostate symptom score, mean storage sub-score, mean voiding sub-score and mean quality of life sub-score were evident after 4-weeks of treatment with further improvements after 8-weeks. At the end of the treatment, all patients declared that their condition improved enough to spare or delay surgery**.**

**Conclusions:**

Silodosin 8 mg once daily significantly improves benign prostatic obstruction in Caucasian patients with lower urinary tract symptoms suggestive of benign prostatic enlargement waiting for surgery.

**Trial registration:**

EudraCT n. 2015-002277-38 Date of registration: 15th December 2017.

## Background

Silodosin is a new, highly selective α1-blocker (AB) approved in Japan in 2006 and recently in more than 50 countries including United States and Europe for the treatment of Lower Urinary Tract Symptoms suggestive of Benign Prostatic Enlargement (LUTS/BPE) [1]. This agent has a very strong affinity for the AR, the predominant α1A Adrenergic Receptor (α1-AR) subtype expressed in human prostate where it mediates smooth muscle contraction and therefore functional obstruction of the lower urinary tract [2–7]. Phase III randomized controlled trials as well as post hoc analyses of these studies performed in Japan, US and Europe demonstrated that silodosin provides clinically relevant benefits in terms of storage and voiding LUTS as well as in terms of Quality of Life (QoL) as assessed by the International Prostate Symptom Score (IPSS) [8–11]. Benign Prostatic Obstruction (BPO) is considered a key pathophysiological link between Benign Prostatic Enlargement (BPE) and LUTS [12, 13]. Moreover, long lasting BPO may activate pathways leading to progressive remodeling of both lower and upper urinary tract with subsequent functional impairments [12]. Therefore, BPO relief represents a major goal of LUTS/BPE treatment. A diagnosis of BPO requires an invasive Pressure/Flow studies (PFS) that allows to calculate the Bladder Outlet Obstruction Index (BOOI) [12]. Clinical studies investigating invasive urodynamic measures of BPO in LUTS/BPE patients receiving silodosin demonstrated that this agent, as other ABs, significantly improves BOOI [11–13]. Based on indirect comparisons, the magnitude of BPO improvement with silodosin appears to be greater if compared to other ABs [12–14]. Until now, however, urodynamic data on silodosin in terms of BPO mainly derive from three major studies involving Japanese LUTS/BPH patients [15–17]. It is widely recognized that ethnic and even population differences exist in the pharmacokinetics and pharmacodynamics of drugs [18]. Moreover, in two of these studies, silodosin was administered at the dosage of 4 mg twice daily whereas the dose of silodosin recommended by both the Food and Drug Administration and EMA is 8 mg once a day [15, 17, 19]. We aimed to preliminary investigate the effects of silodosin 8 mg once daily in Caucasian patients with LUTS/BPE in terms of invasive BPO urodynamic parameters and subjective symptoms.

## Methods

We performed a single-center, open-label, single-arm, post-marketing interventional clinical trial (EudraCT n. 2015-002277-38). The local ethics committee approved the study protocol. The study was carried out according to the Declaration of.

Helsinki. All patients enrolled complained with LUTS severe enough to require surgery and reported poor results with previous pharmacological treatments.

Study inclusion criteria were: Caucasian subjects aged ≥50 years waiting to undergo surgical intervention for LUTS/BPH, IPSS total score ≥ 13, IPSS-QoL score ≥ 3, prostate volume ≥ 30 ml, maximum urine flow rate (Qmax) ≤ 15 mL/s, BOOI > 40. Exclusion criteria were: pharmacological treatment for LUTS/BPH in the last 4 weeks or 6 months in case of previous assumption of 5alpha-reductase inhibitors, absolute indication for surgery therapy, hypersensitivity to the active substance or to any of the excipients, neurological causes of detrusor overactivity, active urinary tract infections, presence or history of bladder calculi, presence of prostate cancer, Post-Void Residual Volume (PVR) > 300 mL, clinically significant cardiovascular and cerebrovascular disease within 6 months prior to screening, renal or hepatic impairment, patients for whom cataract surgery was scheduled, history of orthostatic hypotension or syncope. After a washout period of 6 months, in patients taking 5-alpha reductase inhibitors, or of 4 weeks in patient taking any other drug or herbal remedy for LUTS/BPH, four visits were foreseen: at screening (Visit 1, week − 1), at baseline (Visit 2) and after 4 (Visit 3) and 8 (Visit 4) weeks of treatment. Screening procedures consisted of: medical history collection, check of prior and concomitant medications, symptom assessment by IPSS questionnaire, physical examination, measurement of vital signs (sitting blood pressure and heart rate), evaluation of prostate volume and PVR by suprapubic ultrasound, measurement of Q_max_, 12-lead ECG, laboratory tests, including Prostate Specific Antigen value. Each patient signed an informed consent. Inclusion and exclusion criteria were preliminarily evaluated. At baseline, the following evaluations were performed: vital signs, urodynamic study. A final evaluation of inclusion and exclusion criteria was performed at this stage and patients with a BOOI< 40 were excluded. Eligible subjects received one capsule of silodosin 8 mg once daily for 8 weeks. They agreed not to use any other approved or experimental medication for LUTS/BPE or overactive bladder anytime during the study. At the end of the 4-weeks treatment period, patients underwent the following evaluations: IPSS questionnaire and patient-reported Treatment-Emergent Adverse Events (TEAEs) evaluated based on the terminology of the Medical Dictionary for Regulatory Activities. At the end of the 8-weeks treatment period, patients underwent the following evaluations: physical examination, IPSS questionnaire, vital signs, laboratory tests, PVR, urodynamic study. Moreover, TEAEs were evaluated again. All urodynamic studies were performed by the same operator based on standard International Continence Society (ICS) procedure [20]. A 6-F double lumen catheter was inserted transurethrally, and a balloon catheter was inserted from the anus to measure abdominal pressure. The test was done with the patient standing. Physiological saline solution was injected into the bladder at 50 ml per minute after evacuating the bladder. Intravesical pressure, abdominal pressure and detrusor pressure in the storage phase were simultaneously measured and recorded. Detrusor pressure was measured by electrically subtracting the abdominal pressure from the intravesical pressure. Detrusor Overactivity (DO) was defined as involuntary detrusor contractions during the filling phase which may be spontaneous or provoked. At maximum cystometric capacity the pressure/flow study was performed. The BOOI was defined as Detrusor Pressure at Q_max_ (PdetQ_max_) - 2Q_max_ [21]. According to BOOI value, subjects were classified as obstructed (BOOI > 40), equivocal (BOOI 20 − 40), or unobstructed (BOOI < 20) [21]. The primary objective of the study was to evaluate BOOI variations with respect to baseline. The followings were considered secondary outcomes: variations of other urodynamic parameters, improvement from baseline in obstruction class on the ICS BOOI nomogram, PVR variations, IPSS variations, percentage of subjects considering their condition improved enough to spare or delay the surgical intervention for LUTS/BPE, safety profile of the drug. Continuous variables were expressed as mean ± standard deviation (SD) and categorical variables as number and percentages. Changes from baseline for continuous data were compared using the paired Student’s T test. McNemar’s or Bowker’s Symmetry tests were used for shift tables assessment *P* values of less than 0.05 were regarded as statistically significant. Statistical analysis was performed using SAS software (SAS Institute Inc., Cary, N.C.).

## Results

Overall, 34 subjects were screened. Of them, 4 were excluded after PFS indicated that they were unobstructed. Demographic and clinical characteristics of the 30 subjects enrolled into the study are summarized in Table 1.

**Table 1:** Baseline patients’ characteristics

Demographics	
Age, yr., mean (SD)	63.1 (9.2)
Age category, n (%)	
< 65 yr	16 (53.3)
65–74 yr	10 (33.3)
≥ 75 yr	4 (13.3)
Race, n (%)	
White	30 (100)
Clinical characteristics	
Body Mass Index, kg/m^2^, mean (SD)	24.7 (1.8)
Time elapsed from the diagnosis of LUTS/BPE (yr), mean (SD)	6.0 (3.9)
Prostate volume mL, mean (SD)	58.2 (17.4)
PVR volume, mL, mean (SD)	71.1 (33.1)
Q_max_, mL/s, mean (SD)	9.0 (2.7)
Prostate Specific Antigen, ng/mL, mean (SD)	2.3 (1.8)
Comorbidities, n (%)	
Hypercholesterolemia	1 (3.3)
Asthma	1 (3.3)
Essential hypertension	4 (13.3)

*SD* Standard Deviation

**Fig. 1:**
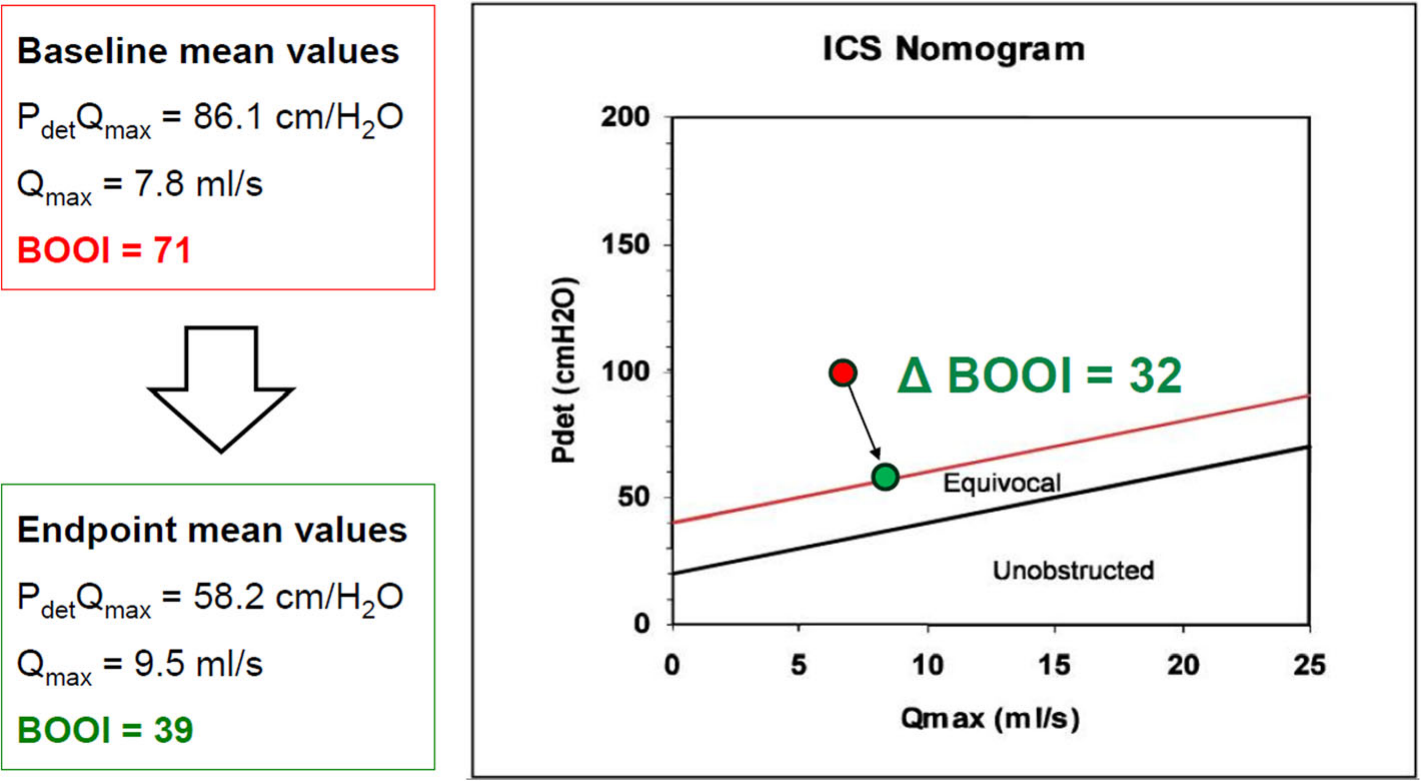
Baseline and endpoint mean urodynamic parameters

All patients completed the study protocol and were available for follow-up evaluations. Table 2 and Fig. 1 summarizes the variations of main urodynamic parameters from baseline to the end of the study.

**Table 2:** Urodynamic parameters at baseline and after 8 weeks of therapy

	Baseline	8 weeks	*P* value
Volume at first desire to void, mL, mean (SD)	105.7 (36.6)	130.3 (42.5)	0.004^‡^
Maximum cystometric capacity, mL, mean (SD)	229.7 (70.0)	257.5 (71.2)	0.0717^‡^
Bladder compliance (mL/ cmH_2_O), mean (SD)	17.2 (27.7)	26.5 (38.9)	0.2851^‡^
DO, n (%)	4 (13.3)	2 (6.7)	0.3173^†^
Amplitude of the largest DO contraction, cmH_2_O, mean (SD)	9.0 (25.0)	4.3 (12.1)	0.3098^‡^
Pdet Q_max_, cmH_2_O, mean (SD)	86.1 (19.7)	58.2 (17.3)	< 0.0001^‡^
Pdet_max_, cmH_2_O, mean (SD)	99.6 (23.5)	72.0 (22.4)	< 0.0001^‡^
Detrusor opening pressure, cmH_2_O, mean (SD)	72.1 (31.0)	50.3 (23.1)	0.0031^‡^
Qmax, mL/s, mean (SD)	7.8 (3.1)	9.5 (3.8)	0.015^‡^
BOOI, mean (SD)	70.6 (18.9)	39.2 (18.3)	< 0.0001^‡^
BOOI< 20 n, (%)	0 (0)	4 (13.3)	
BOOI 20–40 n, (%)	0 (0)	12 (40.0)	
BOOI> 40 n, (%)	30 (100)	14 (46.7)	
Bladder Contractility Index, mean (SD)	121 (34.7)	105.6 (26.5)	0.0274^‡^
PVR volume, mL, mean (SD)	71.1 (33.1)	52.5 (23.2)	< 0.0001^‡^

*BOOI* Bladder Outlet Obstruction Index, *DO* Detrusor Overactivity, *Pdet*_*max*_ Maximum Detrusor Pressure, *PdetQ*_*max*_ Detrusor Pressure at Q_max_, *PFS* Pressure/Flow Study, *PVR* Post-Void Residual Volume, *Q*_*max*_ Maximum urine flow rate, *SD* Standard Deviation

^†^Mc Nemar’s Test

^‡^paired T Test

Mean BOOI significantly decreased from 70.6 to 39.2 and BOOI class improved in 16 patients (53.3%). Obstruction persisted in 14/30 subjects (46.7%). Statistically significant improvements were observed in terms of Detrusor opening pressure, PdetQ_max_, Maximum Detrusor Pressure (Pdet_max_), Q_max_, and Bladder Contractility Index. No statistically significant variations in terms of incidence of DO and amplitude of the largest DO contraction were observed. Total IPSS score, IPSS storage and voiding sub-scores as well as IPSS QoL sub-score improved in a statistically significant manner after 4-weeks of treatment and further improvements were evident after 8-weeks (Table 3).

**Table 3:** IPSS scores at baseline, after 4 weeks and after 8 weeks of treatment

	Baseline	4 weeks		8 weeks	
	Mean (SD)	Mean (SD)	*p* ^†^	Mean (SD)	*p* ^†^
Total IPSS score	21.6 (3.1)	14.9 (3.6)	< 0.0001	10.9 (2.2)	< 0.0001
IPSS storage sub-score	8.3 (1.8)	6.2 (1.5)	< 0.0001	4.4 (1.0)	< 0.0001
IPSS voiding subs-core	13.2 (2.2)	8.7 (2.4)	< 0.0001	6.5 (1.5)	< 0.0001
IPSS-QoL sub-score	4.6 (0.8)	2.9 (0.8)	< 0.0001	1.5 (0.7)	< 0.0001

*IPSS* International Prostate Symptom Score, *SD* Standard Deviation, *QoL* Quality of Life

^†^with respect to baseline (Paired T Test)

Mean total IPSS score improved by 6.7 points and by 10.7 points after 4 and 8 weeks of treatment, respectively. Mean IPSS storage sub-score improved by 2.2 points and 4.0 points after 4 and 8 weeks of treatment, respectively. Mean IPSS voiding sub-score improved by 4.5 points and 6.7 points after 4 and 8 weeks of treatment, respectively. Mean IPSS QoL sub-score improved by 1.7 points and 3.0 points after 4 and 8 weeks of treatment, respectively. At the end of the treatment all patients answered “yes” to the question: “After the treatment with silodosin, is your condition improved enough to spare or, delay the surgical intervention for BPH/LUTS?”. In total, 11/30 patients (36.7%) experienced TEAEs. All TEAEs were drug related. The most frequently reported TEAE was retrograde ejaculation occurring in 8/30 patients (26.7%). Other TEAEs were asthenia (1/30, 3.3%), fatigue (1/30, 3.3%), and nasal congestion (1/30, 3.3%). There were no serious adverse events and all adverse evets were of mild intensity. There were no TEAEs leading to drug discontinuation. No clinical changes were found in terms of vital signs and laboratory parameters.

## Discussion

ABs aim to inhibit the effect of endogenously released noradrenaline on smooth muscle cells in the prostate and thereby reduce prostate tone and BPO [21, 22]. To date, six ABs (terazosin, doxazosin, tamsulosin, naftopidil, alfuzosin, and silodosin) have been approved for the treatment of LUTS/BPE. Although all ABs improve BPO, the magnitude of improvement varies according to the type of AB and is greater after silodosin with values comparable to that obtained after transurethral microwave thermotherapy [12, 13]. To our knowledge, this is the first study investigating the urodynamic efficacy of silodosin 8 mg once a day in terms of BPO in Caucasian patients with LUTS/BPE waiting for surgery. We demonstrated that silodosin significantly improves BOOI in this selected subset of patients characterized by significant subjective and objective impairments. The magnitude of BOOI improvement (31.4 points) was both statistically and clinically significant and in line with published ranges for silodosin (21.2 - 37.6). To date, the rationale behind the urodynamic profile of silodosin is not completely understood. Based on published data, however, the existence of a positive relationship between α-1A/ α-1B receptor affinity ratio and BPO improvement has been hypothesized [12]. Moreover, the magnitude of BOOI improvement with ABs has been reported to increase also with the percentage of patients with obstruction at baseline [13]. Nevertheless, direct comparisons among ABs in terms of BPO improvement are lacking thus limiting the value of current evidences. We found that obstruction class improved in 53.3% of patients. This finding is in line with published data. In the study by Yamanishi et al., the obstruction grade was improved in 15 patients (56%) (obstructed to unobstructed in 5, obstructed to equivocal in 8, and equivocal to unobstructed in 2) and unchanged in 12 (44%) (obstructed to obstructed in 9, and equivocal to equivocal in 3) [16]. The BOOI value is obtained from Q_max_ and PdetQ_max_ values. Results from the present study show statistically significant improvements of both PdetQ_max_ and Q_max_. However, although mean PdetQ_max_ variation was clinically robust, mean Q_max_ improvement was clinically marginal (only 1.8 mL/s). This finding is coherent with published urodynamic data obtained in LUTS/BPE patients treated with ABs in general as well as in the subgroup of Japanese patients treated with silodosin at the dosages of both 4 mg twice daily and 8 mg once a day [13–17]. From a pathophysiological point of view, we can hypothesize that the reduction of detrusor pressure represents a priority with respect to urinary flow improvement and, when the relief of outflow resistances is small as after therapy with ABs, the lower urinary tract mainly adapts by reducing detrusor pressures thus potentially preserving the integrity of the bladder itself and of the upper urinary tract [12]. Therefore, in everyday clinical practice, Q_max_ improvement alone may underestimate the urodynamic benefits deriving from ABs therapy. Unlike some published data, our study did not demonstrate statistically significant variations of urodynamic parameters relative to DO [15–17]. However, the number of patients with DO at baseline in the present study was very low and further studies are needed to specifically address this issue.

In line with published data, we observed a statistically and clinically significant therapeutic effect, including improvements in both obstructive and irritative sub-scores, which was reflected by improvements in QoL. Interestingly, the magnitude of improvement we found in all these domains after 8 weeks of treatment was higher if compared to results from phase III clinical studies on silodosin as well as to results obtained in the urodynamic studies involving mainly obstructed patients after a 12 weeks treatment period [1, 15–17]. Matsukawa et al. reported mean improvements of total IPSS, voiding IPSS sub-score, storage IPSS sub-score and QoL sub-score of 6.2, 3.6, 2.6, and 1.6 points, respectively [17]. Yamanishi et al. reported mean improvement of total IPSS, voiding IPSS sub-score, storage IPSS sub-score and QoL sub-score were 7.9, 3.8, 2.0, and 1.1 points, respectively [16]. In the study by Chapple et al., mean improvement of total IPSS, voiding IPSS sub-score, storage IPSS sub-score and QoL sub-score were 7.0, 4.5, 2.5, and 1.1 points, respectively [10]. The rationale behind the differences observed between our results and published ones is unknown. Although we can hypothesize that baseline characteristics of subjects may have a role and that subjects with a moderate-to-high compromised baseline level, such that involved into the present study, may have a greater margin of improvement if compared to subjects that are less compromised at baseline, further studies are needed to confirm this hypothesis. At the end of the treatment, all patients declared that their condition had improved enough to spare or delay surgery. Results from the present study have relevant clinical implications. Indeed, therapy with ABs may represent an interesting “rescue treatment” option for patients with BPO waiting for surgical treatment.

Although a timely indication to surgery is crucial to prevent bladder decompensation leading to surgical failure, this treatment may postpone the need to perform a surgical treatment in patients showing an improvement of BPO and LUTS and/or an improvement of their QoL due to LUTS while waiting for surgery. Moreover, this treatment may cause a reduction in the waiting list for the other patients confirming the need to perform surgery. The urodynamic profile of silodosin characterized by the highest level of BOOI improvement with respect to other ABs, makes it a drug of greatest interest in this subset of patients. We confirmed the good safety profile of silodosin. In line with published data, retrograde ejaculation was the more frequently reported adverse event. However, none of subjects interrupted the treatment due to this event. We acknowledge potential limitations of the present study. First, it was a single arm, open-label study using neither a placebo nor a control group. Although a placebo-effect cannot be excluded in terms of changes of both subjective and urodynamic parameters, results from a previous meta-analysis suggested the absence of significant placebo effects on urodynamic parameters of BPO after therapy with ABs [13]. Moreover, our study sample voluntarily included patients with urodynamic proven BPO thus making questionable the generalizability of our results to subjects that in the everyday clinical practice do not routinely undergo invasive urodynamic investigations. However, the combination of the clinical criteria we adopted in the pre-screening procedure (age ≥ 50 years, IPSS total score ≥ 13, prostate volume of ≥ 30 ml, Q_max_ ≤ 15 mL/s) represented a good proxy of BPO as it allowed us to identify a population of subjects with a high prevalence of urodynamic proven BPO (88.2%, *n* = 30/34). Therefore, in every day clinical practice, findings from the present study can be generalizable to subjects with the aforementioned clinical features even in the absence of urodynamic confirmation of BPO. Overall, results from the present study should be considered as preliminary and confirmatory randomized placebo controlled trials with adequate follow-up are needed. In conclusions, silodosin 8 mg once daily provides statistically and clinically significant improvement of BPO and symptoms in LUTS/BPE Caucasian men with confirmed BPO waiting for surgery.

## Conclusions

In conclusions, based on these preliminary data, silodosin 8 mg once daily significantly improves BPO in Caucasian patients with LUTS/BPE waiting for surgery.

## Abbreviations

AB: Alpha Blocker
AR: Adrenergic Receptor
BOOI: Bladder Outlet Obstruction Index
BPE: Benign Prostatic Enlargement
BPO: Benign Prostatic Obstruction
DO: Detrusor Overactivity
ICS: International Continence Society
IPSS: International Prostate Symptom Score
LUTS: Lower Urinary Tract Symptoms
PdetQ_max_: Detrusor Pressure at Q_max_
PFS: Pressure/Flow studies
PVR: Post-Void Residual Volume
Q_max_: Maximum Urine Flow Rate
QoL: Quality of Life
SD: Standard Deviation
TEAEs: Treatment-Emergent Adverse Events
